# Supplementary material for: Cloud BioLinux: pre-configured and on-demand bioinformatics computing for the genomics community
Source: BMC Bioinformatics. 2012 Mar 19;13:42. doi: 10.1186/1471-2105-13-42 (PMC3372431; doi:10.1186/1471-2105-13-42)
Supplement: Additional file 1 — Supplementary 1 Cloud BioLinux software documentation in the form of a mini, self-contained website. Users need to download and uncompress the .zip file, and open through a web browser the "index.html" file available on the main directory. (ZIP 1823 kb). [file 1471-2105-13-42-S1.ZIP › Cloud-BioLinux-Package-Documentation/docs/Rcross.html]

Bio-Linux Software Documentation Pages

Back to search form

## Rcross

|  |  |
| --- | --- |
| Name | Rcross |
| Description | **Rcross** is part of the QTL Cartographer suite of programs.  **Rcross** performs a random cross or reformats a data set. It simulates marker and trait data and can also translate files from three different formats. If the user chooses to translate a file, then the simulation options are ignored. |
| Homepage | http://statgen.ncsu.edu/qtlcart/Rcross.php |
| Remote Documentation | http://statgen.ncsu.edu/qtlcart/Rcross.php |

Performs a random cross or reformats a data set.
